# Perspectives on miRNAs Targeting DKK1 for Developing Hair Regeneration Therapy

**DOI:** 10.3390/cells10112957

**Published:** 2021-10-30

**Authors:** Dimitri Papukashvili, Nino Rcheulishvili, Cong Liu, Fengfei Xie, Deependra Tyagi, Yunjiao He, Peng George Wang

**Affiliations:** School of Medicine, Southern University of Science and Technology, Shenzhen 518000, China; dimitri@sustech.edu.cn (D.P.); nino@sustech.edu.cn (N.R.); 11930759@mail.sustech.edu.cn (C.L.); xieff@mail.sustech.edu.cn (F.X.); deependrat@sustech.edu.cn (D.T.)

**Keywords:** miRNA, AGA, DKK1, Wnt/β-catenin, hair

## Abstract

Androgenetic alopecia (AGA) remains an unsolved problem for the well-being of humankind, although multiple important involvements in hair growth have been discovered. Up until now, there is no ideal therapy in clinical practice in terms of efficacy and safety. Ultimately, there is a strong need for developing a feasible remedy for preventing and treating AGA. The Wnt/β-catenin signaling pathway is critical in hair restoration. Thus, AGA treatment via modulating this pathway is rational, although challenging. Dickkopf-related protein 1 (DKK1) is distinctly identified as an inhibitor of canonical Wnt/β-catenin signaling. Thus, in order to stimulate the Wnt/β-catenin signaling pathway, inhibition of DKK1 is greatly demanding. Studying DKK1-targeting microRNAs (miRNAs) involved in the Wnt/β-catenin signaling pathway may lay the groundwork for the promotion of hair growth. Bearing in mind that DKK1 inhibition in the balding scalp of AGA certainly makes sense, this review sheds light on the perspectives of miRNA-mediated hair growth for treating AGA via regulating DKK1 and, eventually, modulating Wnt/β-catenin signaling. Consequently, certain miRNAs regulating the Wnt/β-catenin signaling pathway via DKK1 inhibition might represent attractive candidates for further studies focusing on promoting hair growth and AGA therapy.

## 1. Introduction

Hair growth is a relatively complex process. The human hair cycle comprises three main phases: anagen, catagen, and telogen [1]. Anagen is an active growing phase of hair and lasts for the longest period (up to eight years). During the catagen phase (2–3 weeks), hair follicles (HF) undergo regression. This is followed by the telogen, which is the resting phase (~3 months) [2]. Ultimately, hair shedding takes place, that may be considered as the fourth phase—exogen [3,4].

Hair growth is controlled and influenced by various endogenous factors, including intracellular and intercellular signaling molecules. Some of the influencing factors have inhibitory and some stimulatory effects on the hair cycle. The initiation of the anagen phase is inhibited by the activation of bone morphogenetic proteins (BMP) as well as transforming growth factor β (TGF-β) signaling [2]. On the other hand, the onset of Wnt/β-catenin [5,6,7] or sonic hedgehog (SHH) signaling pathways stimulate hair growth and anagen entry [2,8]. In case of the wingless and integrated-1 (Wnt)-mediated hair regrowth, hypo-phosphorylated β-catenin is stabilized, which triggers the interaction between β-catenin and T-cell factor/lymphoid enhancer factor (TCF/LEF) in the nucleus. As a result, growth-promoting genes are trans-activated and hair regrowth occurs [2]. Based on the available data, out of all the pathways implicated in the hair cycle, we assume that the Wnt/β-catenin signaling represents the key factor in hair growth regulation [9], as dihydrotestosterone (DHT)—a hormone that is upregulated in androgenetic alopecia (AGA) and causes the hair loss—impairs this signaling pathway [10]. The main identified inhibitors of this signaling pathway are dickkopf-related protein 1 (DKK1), secreted frizzled-related protein 2 (SFRP2), and sclerostin (SOST). As Wnt/β-catenin signaling is involved in numerous biological processes, each of the mentioned inhibitors features certain functions. Hence, the dysregulation of the levels of any of these inhibiting proteins affects the particular physiological pathogenesis, including AGA. DKK1 is a natural inhibitor of Wnt, and strongly suppresses the Wnt/β-catenin signaling pathway via disrupting the Wnt-induced frizzled-low-density lipoprotein receptor-related proteins (LRP) 5/6 complex formation. This adversely impacts HF morphogenesis and, thus, influences the hair cycle [11]. Indeed, the study has demonstrated that DKK1 triggers anagen-to-catagen transition when injected in the skin of C57BL/6 mice. Moreover, injection of the anti-DKK1 neutralizing antibody resulted in the delay of catagen progression [12]. Interestingly, the human study showed that a significantly high concentration of tissue DKK1 was present in patients with AGA compared to the healthy controls [11]. DHT is known to negatively interfere with the normal hair cycle via driving the HF in the balding scalp to catagen entry and inhibiting the growth of keratinocytes that takes place through DKK1 implication [13]. 

According to the aforementioned evidence, it is prudent to postulate that inhibition of DKK1—a canonical Wnt/β-catenin signaling inhibitor—is one of the key factors to promote hair growth and develop a sustainable remedy for AGA. Indeed, there are certain microRNAs (miRNAs) that target DKK1 and are involved in hair growth-related pathways. Thereby, in this review, the possible application of miRNAs for AGA therapy via targeting upregulated DKK1 expression in the balding scalp and activating Wnt/β-catenin is postulated.

## 2. The Importance of Wnt/β-Catenin Signaling in Hair Growth

The canonical Wnt/β-catenin signaling is one of the most studied pathways in biology as it is responsible for a number of vital physiological processes in the body [14]. Controlling the cell fate, including cell proliferation, differentiation [15], normal functioning, apoptosis [16], and maintaining tissue homeostasis, as well as affecting cancer development [15,17,18,19], are among them [15,17,20,21,22,23]. Interestingly, apart from the mentioned involvements in various physiological conditions, dysregulation of Wnt/β-catenin signaling is strongly associated with the development of AGA—a dermatological disorder whereby the hair is abnormally shed from the skin where it normally should be presented. Particularly, it is suppressed in AGA [13,22,24,25,26,27]. Indeed, various Wnt proteins play a substantial role in the hair growth cycle [2]. Wnt proteins such as Wnt3a [28,29,30] and Wnt10b [6,31] are essential for HF growth and hair regeneration. In this regard, finding the best strategy for reactivation of the Wnt/β-catenin signaling pathway in people with alopecia has emerged as an area of study. For a better understanding of how this pathway functions in normal conditions and AGA, the molecular mechanism is addressed here: The hair starts growing from the bottom of HFs—mini-organs that are integrated in the epidermis and anchor each of the hairs into the skin with the hair bulb, that forms the base of the HF. The HFs play a pivotal role in hair cycling regulation, together with the whole well-organized complex of structures that assembles the HF itself [32,33]. The cells in the hair bulb are divided and form a hair shaft. Hair bulbs surround dermal papilla (DP) cells that are the key components in the molecular crosstalk between the mesenchymal and neighboring epithelial cells during the hair cycle [4]. The Wnt/β-catenin signaling pathway plays an indispensable role in regulating HF morphogenesis during the embryonic stage and adult life [6,22,34,35,36]. 

**Figure 1:**
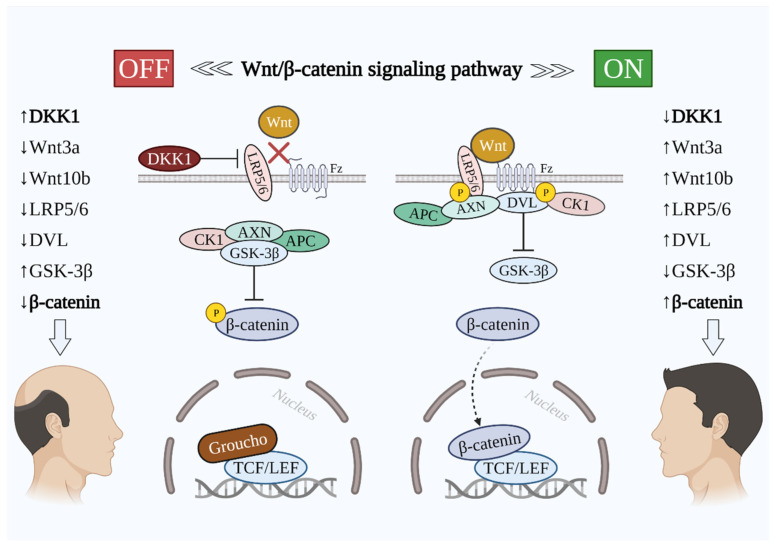
The implication of key molecules in the Wnt/β-catenin signaling pathway in hair loss and growth. Wnt/β-catenin signaling pathway OFF (on the left side): DKK1 inhibits LRP5/6 and does not allow Wnt proteins to activate the signaling pathway. Destruction complex inhibits β-catenin and its translocation into the nucleus is prevented. Transcription of Wnt-targeted genes is hindered. Wnt/β-catenin signaling pathway ON (on the right side): Wnt binds to Fz and LRP5/6, which is followed by the phosphorylation of LRP5/6 intracellularly that leads to the DVL recruitment to Fz. β-catenin is then translocated into the nucleus and displaces Groucho. Transcription of Wnt-targeted genes takes place. Abbreviations: DKK1, dickkopf-related protein 1; Wnt, wingless and integrated-1; Fz, frizzled; LRP5/6, low-density lipoprotein receptor-related proteins 5/6; AXN, axin; GSK3-β, glycogen synthase kinase 3β; CK1, casein kinase 1; DVL, dishevelled; APC, adenomatous polyposis coli; TCF/LEF, T-cell factor/lymphoid enhancer factor.

During the canonical Wnt/β-catenin signaling, Wnt ligands (Wnt1a, 2, 3a, 4, 5a, 7b, 10a, and 10b) bind the frizzled receptor and LRP5/6 co-receptor outside of the plasma membrane [2,16], which induces the phosphorylation of LRP5/6 from the intracellular part of the plasma membrane by two kinases—glycogen synthase kinase 3β (GSK-3β) and casein kinase 1 (CK1)—which are the part of the destruction complex. This is followed by the recruitment of protein dishevelled (DVL), which binds to the frizzled receptor endo-domain. As a result, the destruction complex is inactivated, which protects β-catenin from targeting by the proteasome [37]. Consequently, hypo-phosphorylated β-catenin is stabilized and accumulated in the cytoplasm [16]. Stabilized β-catenin is able to translocate into the nucleus, where it binds to TCF/LEF via displacing the transcriptional repressor Groucho [38]. A transcriptional complex is formed, and the transcription of several Wnt-target genes is activated [16,38]. On the other hand, when Wnt signaling is off, β-catenin is then phosphorylated by GSK-3β and CK1. Except for GSK-3β and CK1, the destruction complex consists of two other components—scaffolding protein AXIN and adenomatous polyposis coli (APC) protein. The phosphorylated β-catenin then undergoes ubiquitination, which makes it a target of the proteasome. When Wnt/β-catenin signaling is off, β-catenin becomes unstable and undergoes proteasomal degradation [37,39]. As a result, the transcriptional complex is not formed, and Groucho remains bound to TCF/LEF. Hence, the gene expression is suppressed [16,38]. When the DKK1 level is upregulated, it binds to LRP5/6, which suppresses the whole cascade of the signaling pathway, leading to the inhibition of HF development and, consequently, hair growth [31] (Figure 1). As previously mentioned, DKK1 overexpression is evidenced in the scalp of patients with AGA. Thus, it represents the key protein in the development of AGA [40].

Although SFRP2 is also considered to be the inhibitor of the Wnt/β-catenin signaling pathway, some conflicting data exist. Kwack et al. have studied the influence of SFRP2 on cultured human DP cells obtained from patients undergoing hair transplantation via punch biopsy. The results showed that treatment with recombinant human SFRP2 remarkably increased Wnt/β-catenin signaling [41]. Thus, DKK1 appears to be the key target molecule for studies that will focus on strategies of Wnt/β-catenin signaling augmentation in DP cells of AGA. Interestingly, a number of miRNAs is involved in the regulation of hair growth [42], and impairment of Wnt/β-catenin signaling negatively affects normal hair cycling [2]. This raises the idea of using certain miRNAs for modulating hair growth via regulating the Wnt/β-catenin signaling pathway.

## 3. DKK1 Implication in AGA 

### 3.1. DHT-Induced DKK1-Mediated AGA

DKK genes encode secreted proteins that regulate the Wnt/β-catenin signaling pathway by antagonizing it [43]. Remarkably, DHT and DKK1 levels are positively correlated. In the body, 5 alpha-reductase (5αR) converts testosterone into DHT. It acts like an inhibitor for the growth of outer root sheath (ORS) cells that disrupts the normal hair growth process. Moreover, the anti-DKK1 neutralizing antibody substantially decreased the inhibition of ORS cells’ growth. As previously mentioned, DKK1 concentration is increased in the bald scalp of AGA patients compared with the haired scalp of AGA patients [10,40]. Besides, DHT-influenced DKK1 augmentation has been demonstrated to enhance the apoptosis of keratinocytes in vitro [10]. It can be presumed that DKK1 plays an important role in AGA development. Indeed, the study demonstrated that DKK1 is involved in AGA pathology. In the study, recombinant human DKK1 treatment triggered the hair cycle to enter the catagen phase earlier than normal in C57BL/6 mice, which resulted in the decrease of HF length. Contrarily, treatment with the neutralizing DKK1 antibody led to the increased HF length and delayed the shift of anagen to catagen. The recombinant DKK1 has inhibited the canonical Wnt/β-catenin signaling pathway that promotes hair growth in normal physiological condition. As a result of Wnt/β-catenin signaling suppression, the anagen phase is shortened and the apoptosis of follicular keratinocytes takes place [12].

### 3.2. Negative Influence of DKK1 on Hair Growth

**Figure 2:**
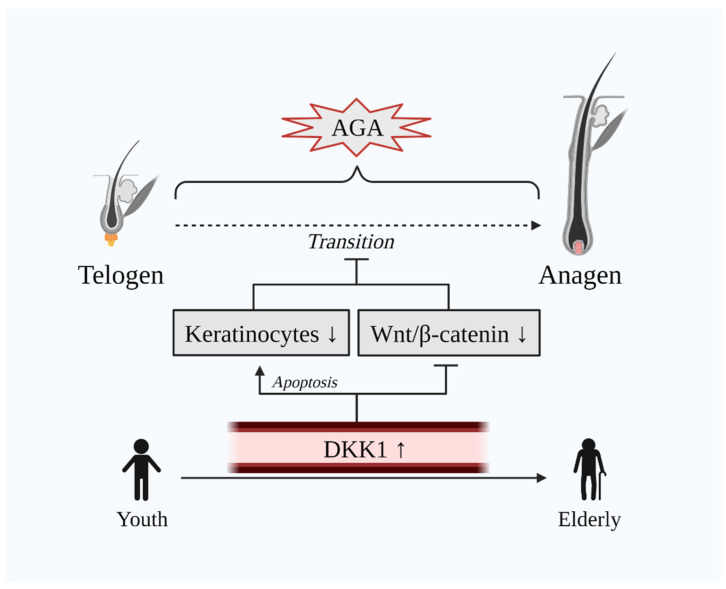
Dual unfavorable role of DKK1 on hair growth. The transition of telogen to anagen is delayed in AGA. DKK1 is upregulated in the scalp of AGA as well as in the serum of the elderly population where AGA is common. Elevated DKK1 levels induce the apoptosis of keratinocytes and inhibit Wnt/β-catenin signaling. Abbreviations: AGA, androgenetic alopecia; DKK1, dickkopf-related protein 1; Wnt, wingless and integrated-1.

DKK1 exhibits a dual role in the normal hair cycle. On the one hand, DKK1 induces attenuation of the hair growth process by inhibiting Wnt/β-catenin signaling via the LRP5/6 co-receptor and, on the other hand, it promotes apoptosis of keratinocytes, which are key cells involved in hair growth [10,40]. Hence, AGA, also known as male pattern baldness (MPB), is the consequence of the abovementioned dual mechanism of DKK1 in human HFs. Indeed, a case-control study that included 20 male AGA and 20 male alopecia areata (AA) patients has demonstrated that the immunohistochemical expression of DKK1 was remarkably increased in lesional scalp biopsies of both AGA and AA patients [40]. Additionally, DKK1 decreases the HF enlargement and width of the hairs [31]. Markedly, DKK1 levels are evidenced to be elevated along with age [44,45], whilst age is also related to the development of AGA [46] (Figure 2). Besides, AGA is manifested severely in the obese population [47,48]. Concomitantly, DKK1 is hypothesized as a potential biomarker in obesity [49,50]. Additionally, Kim et al. have demonstrated that treatment with minoxidil—a common drug for hair-loss—downregulated DKK1 and TGF-β in human keratinocyte cells [51].

### 3.3. Molecular Mechanism of Wnt/β-Catenin Signaling Inhibition by DKK1

**Figure 3:**
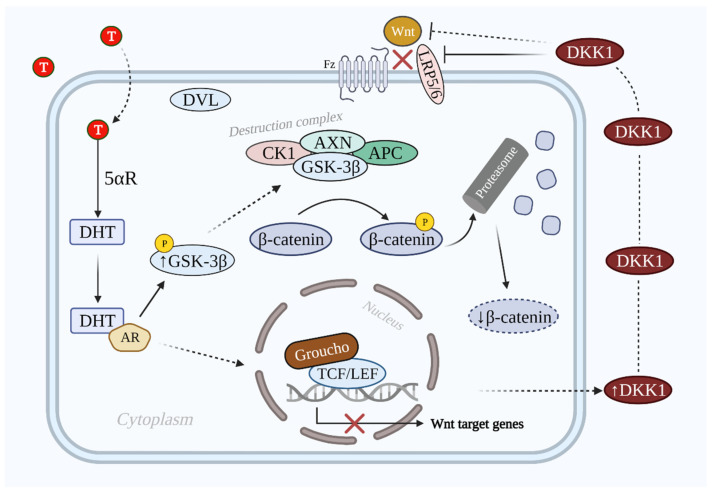
Illustration of Wnt/β-catenin signaling pathway inhibition by DKK1 in AGA. In the AGA population the T level is elevated. T is converted into DHT by 5αR, which binds to AR and prevents the dephosphorylation of GSK-3β. GSK-3β induces the phosphorylation of β-catenin via the destruction complex. Phosphorylated β-catenin is degraded by the proteasome. On the other hand, DKK1 is also secreted that antagonizes Wnt/β-catenin signaling via LRP5/6 inhibition. As a result, the Wnt/β-catenin signaling pathway is inactivated and target genes are not expressed. Abbreviations: T, testosterone; DHT, dihydrotestosterone; 5αR, 5 alpha-reductase; AR, androgen receptor; DKK1, dickkopf-related protein 1; Wnt, wingless and integrated-1; Fz, frizzled; LRP5/6, low-density lipoprotein receptor-related proteins 5/6; GSK-3β, glycogen synthase kinase 3β; CK1, casein kinase 1; DVL, dishevelled; APC, adenomatous polyposis coli; TCF/LEF, T-cell factor/lymphoid enhancer factor.

DKK1 inhibits Wnt/β-catenin signaling via binding to LRP5 and LRP6, which prevents the interaction between Wnt and the other transmembrane receptor frizzled [52]. The inhibition is enhanced via the synergistic effect of DKK1 and its single transmembrane receptors kremens (KRM1 and KRM2) that promote the endocytosis of LRPs. Cselenyi and Lee have proposed that KRMs-dependent activation/inhibition of Wnt/β-catenin signaling depends on the presence of DKK1 [20,53]. In case of DKK1 presence, LRP5/6, DKK1, and KRM interact with each other and generate a complex that is endocytosed. Consequently, LRP5/6 is reduced in the plasma membrane; thus, Wnt/β-catenin signaling is suppressed, β-catenin is degraded, and the hair growth-related gene expression does not take place [54]. The inhibitive role of DHT-induced secreted DKK1 on the Wnt/β-catenin signaling pathway is shown in Figure 3.

### 3.4. Compounds Capable to Inhibit DKK1 Expression and Promote Hair Growth

According to the abovementioned information, the study of DKK1 inhibition strategies is an important tool for maintaining the Wnt/β-catenin signaling within the normal range. Indeed, some studies investigated the effect of certain natural compounds on the canonical Wnt/β-catenin signaling pathway for promoting hair growth. Zhou et al. have observed the impact of morroniside—a natural compound found in cornelian cherry—on the Wnt/β-catenin signaling pathway in cultured human ORS cells. In their results, morroniside indeed increased the proliferation of cells as well as the concentration of β-catenin. Concomitantly, the outcome of morroniside treatment was retrieved by the influence of DKK1 [52]. Another natural compound, vitexin, is also found to significantly increase the human dermal papilla cells’ (HDPCs) proliferation in a concentration-dependent fashion. Additionally, Luo et al. have demonstrated that in vitexin-treated HDPCs, the β-catenin level was upregulated while the DKK1 level was substantially lowered [55]. Aside from that, Panax ginseng extract exhibited proliferation of ORS keratinocytes, inhibition of apoptosis, and revealed the opposite effect of DKK1 in human hair organ culture [56]. One more natural compound costunolide activates Wnt/β-catenin and SHH, while it inhibits TGF-β/SMAD and BMP signaling pathways in HFs, and hence, stimulates hair growth [57]. The functional components of Ginkgo biloba—ginkgolide B and bilobalide—are known as agents that can aid in hair growth. Interestingly, these two compounds are evidenced to be associated with Wnt/β-catenin signaling activation via inhibition of DKK1 expression levels in the cytoplasm and, thus, upregulation of nuclear β-catenin and its messenger RNA (mRNA) concentration [58]. The antidepressant tianeptine is also evidenced to stimulate hair shaft elongation, and hence hair growth, via inhibiting DKK1 and delaying the premature transition from anagen to catagen in MPB [59]. All these studies claim that the abovementioned natural compounds promote hair growth via inhibiting DKK1.

## 4. miRNAs Targeting DKK1 Expression 

miRNA was first identified in 1993 by Victor Ambros and colleagues, who found a short RNA molecule Lin-4 of 22–61 nucleotides in *C. elegans* that could downregulate the level of Lin-14 protein mRNA [60]. Seven years after the discovery, the first human-encoded miRNA Let-7 was described [61]. Currently, more than 3000 miRNAs are known to be present in the human genome and involved in numerous biological processes in almost all body fluids [62,63]. In 2013, the first miRNA mimic MRX34 (mimic of endogenous miR-34 that suppresses oncogenesis) entered into the clinical trials [64]. Currently, several miRNA drugs are in phase 1 or 2 of development [65,66]. However, up until now, there is no miRNA mimic approved by the Food and Drug Administration (FDA) [66]. On the other hand, there are already 3 small (short)-interfering RNA (siRNA)-based FDA-approved drugs, Patisiran [67], Givosiran [68], and Lumasiran [69], while the rest are in phase 3, 2, or 1 of clinical trials [66]. Given that it took 14 years since the initiation of the very first clinical trial (2004) [70] for siRNA to go to commercialization [71,72], it should not be long until the first miRNA mimic gains FDA approval. However, difficulties mainly regarding the delivery of miRNAs need to be overcome and the mechanisms of certain miRNAs should be fully understood. 

Importantly, miRNAs play a crucial role in hair growth regulation [73]. They are involved in HF development as well as in DP cell proliferation. However, despite the increasing number of studies that demonstrate a critical role of miRNAs in skin regeneration, the molecular mechanisms are still not fully understood [74]. Some miRNAs that are implicated in hair morphogenesis have a positive while some have a negative impact on hair growth, e.g., one study showed that miR-214 targets β-catenin and modulates Wnt/β-catenin signaling, and hence inhibits hair growth development [75]. On the other hand, other miRNAs, such as miR-218-5p, are related to inhibition of SFRP2 that represents the antagonist of the Wnt/β-catenin signaling pathway and, thus, promotes hair growth [76,77]. As stated above, the level of DKK1 in the scalp of AGA patients is significantly higher [40]. Besides, DKK1 is already evidenced to inhibit the Wnt/β-catenin signaling, which results in hair miniaturization and growth suppression [78]. There are miRNAs that target DKK1, e.g., miR-335-5p is downregulated while DKK1 protein levels are increased in TNF-α-treated osteoblasts [79]. DKK1 is silenced by miRNAs including miR-335-5p at an early stage of osteogenic differentiation, and on the contrary, miRNA levels are decreased at a later stage of differentiation while DKK1 levels are upregulated. This points out the complex role of miRNAs in biological processes [80]. Besides, Michel et al. have demonstrated that the expression of Wnt antagonist SFRP2 mRNA was increased while the expression of another inhibitor DKK1 mRNA was not altered in scalp biopsies of AGA patients [81]. This fact might be rationalized with the involvement of certain miRNAs that target DKK1 mRNA, and the translation to protein is circumvented. Accordingly, developing the miRNA-based approach to inhibit DKK1 is certainly reasonable. The miRNAs that inhibit DKK1 expression in different health conditions/biological processes are presented in Table 1.

## 5. Available and Recently Studied Therapeutics for AGA

Up until now, there are no clinical trials regarding miRNA therapy for hair growth. To develop the miRNA-based drug for AGA treatment, the appropriate miRNA candidates should be carefully selected, which is the main purpose of this manuscript. Before getting straight to that point, other available approaches are also discussed. Notably, there was no commercially available mRNA-based vaccine before the COVID-19 outbreak. However, effective mRNA vaccines that are being widely used nowadays are hailed as a milestone in vaccinology. Similarly, miRNA-based drugs may become another breakthrough in drug development in the near future. Moreover, some miRNAs are already in clinical trials [65].

Among the currently accessible methods of AGA treatment, transplantation is considered to be the conventional technique, e.g., platelet-rich plasma therapy, follicular unit extraction, and strip harvesting. However, the effectiveness still needs to be studied properly [119]. In several topical and oral medications, minoxidil has already been widely used as a hair loss remedy. Although the exact mechanism of its action is still elusive [120,121], it is evidenced that minoxidil increases the levels of factors that play major roles in hair growth [51], e.g., Wnt5α and vascular endothelial growth factor (VEGF) [122], β-catenin activity, and expression in DP cells [123], while its action results in the downregulation of DKK1 in HDPCs [51]. According to Choi et al., minoxidil promotes the hair growth and proliferation of DP cells via stimulating the release of growth factor from adipose-derived stem cells [124]. Despite its broad application and effects, this medication still exhibits some side effects [121]. Another orally and topically available agent finasteride reduces DHT levels, but has adverse sexual side effects [125,126]. Similar side effects are displayed when oral dutasteride is applied [125,127]. Finasteride [128] and dutasteride [129,130] inhibit 5αR, and thus hinder the production of DHT from testosterone [131]. Despite the side effects, topical minoxidil and oral finasteride are already approved by the FDA and remain the key therapeutics up until now [132]. Low-level laser therapy (LLLT) also seems to improve hair regrowth in the conditions of AGA, AA, and chemotherapy-induced alopecia [133]. There are studies demonstrating the positive effects of natural bioactive compounds on AGA [24,30,52,55,56,58,134,135,136,137,138]. A recently published study demonstrates that a 5-mer peptide (GLYYF; P5) has the potential to promote hair growth when topically applied [139]. Remarkably, stem-cell-based therapy for AGA is also being researched [140]. Aiming to renew the damaged HFs via replacing them with in vitro reconstructed HFs seems promising. Nevertheless, certain issues hamper this approach to be developed [141]. Cholesterol-modified siRNA has also been evidenced to be worthy for topical application for alopecia treatment [142]. Interestingly, the exosomes—membrane-bound extracellular vesicles that are functionally pleiotropic nanoparticles—have also demonstrated their positive role on hair growth in cultured HF [77,140,143]. Exosomes carry exosomal cargos that contain various miRNAs together with other nucleic acids, proteins, lipids, etc. [144]. Thus, although it may be considered as a future approach, the strategy of exosome application for hair growth needs to be carefully developed as the interplay of exosomal miRNAs and other molecules is very complex.

## 6. miRNA Involvement in Hair Growth Regulation

miRNAs are already evidenced to play a substantial role in HF development. The functions of miRNAs are very diverse. The same miRNA has the ability to regulate multiple gene expression. Except for hair growth, miRNAs are related to having a strong influence on osteogenesis, the wound-healing process, tumorigenesis, etc. miRNAs are considered strong gene regulators as they control gene expression. Primary miRNA (pri-miRNA)—the initial form of these small non-coding RNA molecule—is transcribed from introns, then cleaved by an enzyme, Drosha [145]. As a result, a precursor miRNA (pre-miRNA) is formed, which is translocated from the nucleus into the cytoplasm by an exportin-5. In the cytoplasm, it is further processed, the terminal loop is removed by the enzyme Dicer, and mature miRNA duplex formation takes place. It is then recognized by the Argonaute (AGO) protein family that is the part of the RNA-induced silencing complex (RISC), and the guide strand of miRNA is loaded into RISC while the passenger strand is unloaded and degraded [146]. The remaining ~22 nucleotide-length miRNA is capable to bind the target region of specific mRNA on the 3′ untranslated region (3′ UTR) and induces silencing via deadenylation and decapping and, as a result, post-transcriptional regulation of gene expression occurs [147]. The seed region—about 2–8 nucleotides of the 5′ end—determines the specificity of the miRNA guide strand to the 3′ UTR of target mRNA [148]. However, the miRNA–target interaction is not conserved between certain species [148].

Since the first identification of miRNAs in the human body, the link between miRNAs and hair growth regulation was also soon discovered [149]. However, as yet, there is no ideal miRNA-based strategy for hair regrowth developed as the mechanisms are not fully understood. During the hair growth cycle, the expression of miRNAs varies according to the hair growth phases [150,151]. miRNAs are implicated in the development of skin appendages [73]. Hence, these small nucleic acid molecules seem to be a great therapeutic target for the treatment of AGA.

**Figure 4:**
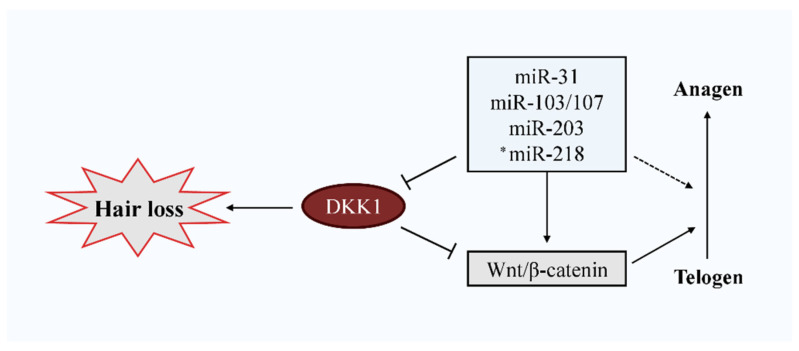
Illustration of potential implication of miRNAs on telogen to anagen transition. miR-31, miR-103/107, miR-203, and miR-218 regulate DKK1 levels and activate the Wnt/β-catenin signaling pathway, which influences telogen to anagen transition. Abbreviations: DKK1, dickkopf-related protein 1. * Indirect inhibition of DKK1 [153].

According to the abovementioned description, hair growth is a complex process that consists of three main phases. Normally, in the telogen phase, hair falls from the scalp and a new anagen starts to induce a new hair growth cycle. In patients with AGA, the transition from the telogen to anagen phase is hindered. The shifting process is controlled by various signaling pathways that can induce or delay transition between phases [152]. miRNAs that play one of the key roles in the hair cycle sensibly merit the attention in molecular biology studies. The possible impact of certain miRNAs on telogen to anagen transition via regulating Wnt/β-catenin signaling pathway is illustrated in Figure 4.

Although their principal function in the body is already evidenced, there are still aspects that need to be elucidated. There are a number of miRNAs that play certain roles in different stages of HF development [73]. Indeed, studies have demonstrated that miR-218-5p promotes hair regeneration in mice via targeting SFRP2 [76,77]. However, in these studies, DKK1 levels were not observed. Basically, the Wnt/β-catenin signaling pathway is a common pathway for different pathophysiological processes, e.g., cancer, where certain miRNAs influence Wnt/β-catenin through inhibiting DKK1 and other involved proteins. Here, we discuss miRNAs that might be implicated in the development of hair growth and that may have a significant outcome in designing miRNA-based sustainable therapy. It is critical to identify the key miRNAs that are essential in hair regrowth to treat AGA.

**Figure 5:**
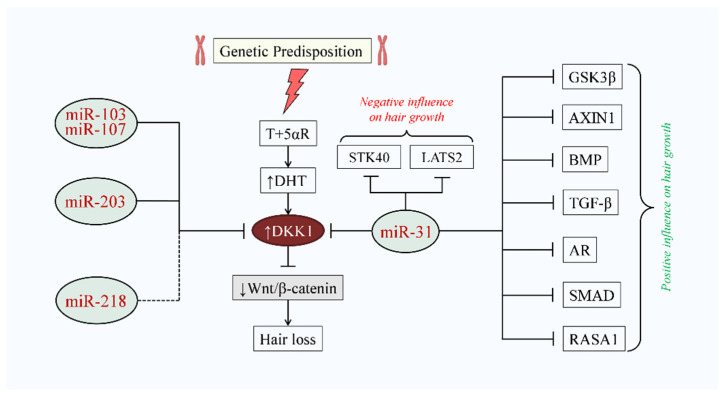
Potential implication of DKK1-targeting key miRNAs in genetically predisposed hair loss. In a genetically predisposed population, T is converted to DHT by 5αR. Upregulated DHT induces DKK1 expression. DKK1 inhibits Wnt/β-catenin signaling and hair loss takes place. miR-103/107, miR-203, and miR-218 reduce the DKK1 level, which might prevent hair growth, while miR-31 potentially inhibits DKK1 and other proteins that might also modulate the hair growth cycle. Abbreviations: T, testosterone; DHT, dihydrotestosterone; 5αR, 5 alpha-reductase; DKK1, dickkopf-related protein 1; GSK-3β, glycogen synthase kinase 3β; STK40, serine/threonine kinase 40; LATS2, large tumor-suppressor kinase 2; BMP, bone morphogenetic protein; TGF-β, transforming growth factor-β; AR, androgen receptor; RASA1, RAS p21 protein activator 1. Dashed line indicates the indirect inhibition of DKK1.

Interestingly, miR-125 is abundantly expressed in balding DP cells [151]. Moreover, it is found to inhibit the receptor of vitamin D, which is required for hair growth [154] as it activates Wnt/β-catenin signaling, among other pathways in keratinocytes [155]. Hence, the inhibition of the vitamin D receptor results in the impairment of hair growth [154]. miR-126 is evidenced to be present in HFs [156], which stipulates that it might be implicated in hair growth regulation. However, there are insufficient studies regarding miR-126. Interestingly, the miR-133b level was abnormally increased in patients with AGA and the levels of β-catenin in HDPCs were decreased, indicating its negative influence on hair growth [150]. The potential influence of key miRNAs in the hair growth process via modulating the Wnt/β-catenin signaling pathway is presented in Figure 5. In addition, Deng et al. have demonstrated that the expression of some miRNAs (miR-133b, miR-141-5p, miR-652-5p, miR-520d-5p, and miR-1247-5p) was markedly upregulated, while the expression of other miRNAs (miR-378d, miR-4286, and miR-3607-5p) was downregulated in the affected region of the AGA group. On the other hand, the expression of miR-133b, miR-141-5p, miR-652-5p, and miR-1247-5p was highly elevated in the affected region of AGA compared with the non-affected region of AGA patients [150]. Broad information of critical miRNAs that may be useful for designing studies on hair morphogenesis is combined in Table 2.

**Figure 6:**
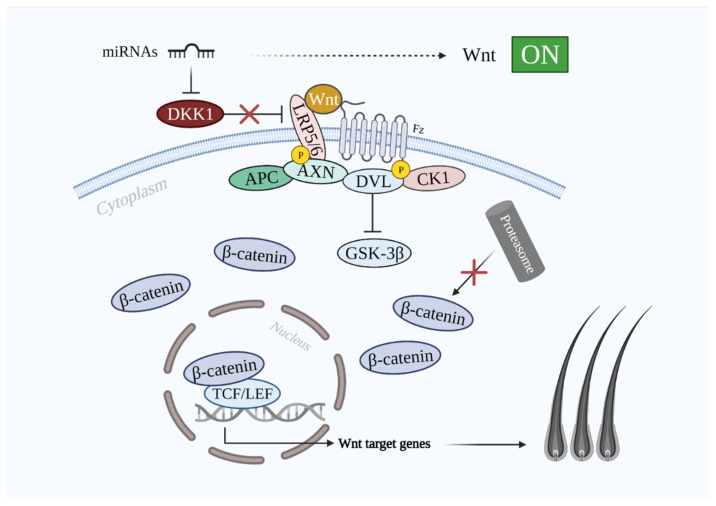
Schematic illustration of miRNAs role in hair growth. miRNAs targeting DKK1 allow Wnt to bind LRP5/6 and Fz to activate the signaling pathway and promote hair growth. Abbreviations: DKK1, dickkopf-related protein 1; Wnt, wingless and integrated-1; Fz, frizzled; LRP5/6, low-density lipoprotein receptor-related proteins 5/6; GSK-3β, glycogen synthase kinase 3β; CK1, casein kinase 1; DVL, dishevelled; APC, adenomatous polyposis coli; TCF/LEF, T-cell factor/lymphoid enhancer factor.

miRNAs are greatly multifunctional agents and the evidence from different sources is often puzzling, e.g., chi-miR-130-5p positively affects the Zhongwei goat hair development [177], while the member of the same miRNA family, miR-130b-3p, negatively influences the hair growth in Cashmere goats [176]. miR-130b-3p targets Wnt10a [176], while according to another study, it targets DKK1 in melanoma cells [91]. Thus, before formulating the suitable miRNA candidates for hair growth regulation, in-depth research is required on each possible target miRNA. The general mechanism of how miRNAs might promote hair growth is provided in Figure 6. miRNAs that are considered to be strongly involved in hair morphogenesis regulation are discussed below.

### 6.1. miR-29

miRNAs are versatile in terms of gene regulation. At the same time, the same miRNA targets a number of proteins. miR-29a is an example of accommodating diverse functions. miR-29s are encoded by two gene clusters and are transcribed by RNA polymerase II. The miR-29 family comprises three mature members: miR-29a, miR-29b, and miR-29c [199]. The seed region, which is a conserved sequence of the miRNA that perfectly binds to the target region of mRNA, is common for miR-29 family members. Hence, the predicted target genes largely overlap. Nevertheless, miR-29s exhibit different regulations, and thus, their functions may be different from each other [199]. These miRNAs accommodate diverse functions. Therefore, they are involved in various health conditions and studied extensively, e.g., miR-29s have elicited both oncogenic and tumor-suppressive functions. Particularly, miR-29b-1-5p was found to be downregulated in human breast cancer tissues, while miR-29b-3p was remarkably overexpressed in the human breast cancer cell line. Moreover, its inhibition was correlated with decreased cell viability, migration, and invasion [200]. 

The studies have demonstrated that miR-29a inhibits the expression of DKK1, KRM2, and SFRP2, and activates Wnt/β-catenin signaling [164,167]. Hsu et al. demonstrated that the gain of miR-29a in diabetic mice is positively correlated with β-catenin levels, and negatively associated with the DKK1 levels, indicating that miR-29a is a regulator of DKK1 and, hence, the Wnt/β-catenin signaling pathway [166]. Indeed, other studies also substantiate the same phenomenon in different sample types [84,164,165]. Based on this, one may presume that miR-29a is implicated in the hair growth cycle, particularly in the promotion of hair growth. In fact, Zhu et al. have demonstrated that miR-29a targets and inhibits the expression of DKK1, KRM2, and SFRP2 in vivo and in vitro. These proteins are involved in the inactivation of Wnt/β-catenin signaling onset. Thus, miR-29a seems to be the candidate for studies focusing on developing AGA treatment strategies [167]. However, according to Ge et al., miR-29a/b1 overexpression inhibits the lineage of mice hair follicle stem cells (HFSCs). Additionally, the authors claim that miR-29a/b1 inhibits LRP, which represses the Wnt/β-catenin signaling and results in hair loss [168]. Hence, the principle of the mechanism still needs to be clarified. Mardaryev et al. have studied the altered expressions of numerous miRNAs in the skin of mice during the different stages of the hair cycle. As a result, miR-29a expression was found to be dramatically downregulated during the anagen phase compared to the telogen phase in mice [170]. Although the abovementioned evidence seems paradoxical, miR-29 might not be excluded as a potentially beneficial agent for hair growth in AGA. Consequently, despite the fact that miR-29a’s impact differs in cell types, its inhibitory effect on DKK1 expression remains unchanged, which leads to the activation of Wnt/β-catenin signaling, and hence, hair growth.

### 6.2. miR-31

miR-31 has a multifunctional capacity as it targets a number of genes and pathways [201]. It is highly expressed in the skin of mice during the anagen phase, compared to the catagen and especially the telogen phase [170]. Remarkably, an elevated level of miR-31 inhibited androgen receptor (AR) expression in vivo [201], which is crucial for ameliorating the AGA condition. Increased AR expression is associated with AGA [202,203]. Kim and Yoon have studied the expression of miR-31 in *Hr^Hp^* (hairless mutant mice, ‘hair-poor’) mice. These mice exhibit hair loss and overexpression of protein hairless. The study demonstrated that the miR-31 level was significantly downregulated in the skin of *Hr^Hp^*/*Hr^Hp^* mice [204]. 

Chen et al. have studied the miR-31-5p regulatory role in osteosarcoma cells via the Wnt/β-catenin signaling pathway. The study demonstrated that miR-31-5p targets AXIN1, which is part of the destruction complex and takes part in the reduction of β-catenin levels, and thus inactivates the canonical Wnt/β-catenin signaling pathway. Thereby, the downregulation of miR-31-5p has an inhibitory effect on the proliferation of osteosarcoma cells via overexpression of AXIN1 [171]. Except for the AXIN1, it targets other factors that are implicated in Wnt/β-catenin signaling, e.g., DKK1 and GSK-3β [205]. Indeed, Lv et al. have also stated that miR-31 activates the Wnt/β-catenin signaling pathway via inhibiting DKK1 in mammary stem cell-enriched mammary basal cell population and in mammary tumors [85]. Apart from activating Wnt/β-catenin signaling, miR-31 inhibits BMP and TGF-β signaling pathways via targeting SMAD3 and SMAD4 in mice [206]. miR-31-5p overexpression is also found to exacerbate the proliferation of goat HFSCs and reduce apoptosis. Feng et al. has demonstrated that in Yangtze River Delta white goats, this regulatory mechanism is conditioned by the capacity of miR-31-5p to suppress RAS p21 protein activator 1 (RASA1) and increase mitogen-activated protein 3 kinase 1 (MAP3K1) levels. Thus, miR-31-5p is also involved in the mitogen-activated protein kinase (MAPK) signaling pathway and, as a result of its mechanism of action, hair growth takes place [169]. On the contrary, Luan et al. have studied the role of miR-31 in the hair growth of a transgenic mouse model and demonstrated that miR-31 impairs hair growth [172]. Additionally, miR-31 upregulation was found to be associated with HF aging in humans [207]. Thus, miR-31 seems to be an arguable candidate for AGA treatment. 

### 6.3. miR-103/107

miR-103 is homologous to miR-107 [208]. miR-107 targets DKK1, while the expression of this Wnt/β-catenin signaling antagonist is negatively correlated with the levels of miR-107, LRP5, and β-catenin in osteosarcoma tissues. It indicates that miR-107 is implicated in the Wnt/β-catenin signaling pathway and may promote its onset [90]. Additionally, miR-103/107 induces downregulation of AXIN2 and enhances the duration of Wnt/β-catenin signaling as well as β-catenin abundance in the nucleus and β-catenin/TCF-dependent reporter activity, which promote multiple stem-like features of colorectal cancer [39]. Moreover, Liu et al. have demonstrated that miR-103a-3p reversed the effect of DKK1 in human bone marrow-derived mesenchymal stem cells [89]. 

These results support the assumption that miR-103/107 may promote hair growth via activating Wnt/β-catenin signaling by targeting DKK1 and AXIN2, and therefore, these miRNAs deserve attention for further studies on AGA. In addition, Wang et al. have demonstrated that miRs-103/107 are expressed in the epidermis and HFs of mice and are downregulated with age. The authors suggest that miR-103/107 may be one of the key factors in sustaining the HF stemness with age [173].

### 6.4. miR-152

Notably, miR-152 with miRNAs 199a, 126, 143, and 214 have been abundantly found in HFs [73,156]. Moreover, miR-152 is among the miRNAs that were found to be overexpressed more than three-fold in the mice HFs [156]. Likewise, Xu et al. have demonstrated that miR-152 levels were inversely correlated to the DKK1 expression level in multiple myeloma cells. Besides, the knockdown of miR-152 resulted in the upregulation of DKK1 mRNA and protein concentration. Moreover, aligning the sequences of miR-152 with the 3′UTR of DKK1 showed 9 binding sites. This, together with the result of transcriptional activity analysis, revealed that miR-152 directly regulates DKK1 gene expression [93]. Concurrently, Zhao et al. have demonstrated that overexpressed miR-152 targets and inhibits DKK1 levels in osteosarcoma cells [209]. It is noteworthy to mention that although miR-152 inhibits DKK1, and it is abundantly expressed in HFs [156], the microarray assay showed the lower levels of mmu-miR-152 expression in anagen and the highest expression in the telogen phase in mice skin [170]. Therefore, it can be presumed that miR-152 may regulate hair growth, but the influence is unclear and needs further investigation.

### 6.5. miR-203

miR-203 is among the most abundantly expressed miRNAs in epidermis [156]. Interestingly, Cheng et al. have suggested that miR-203 targets the 3′UTR of DKK1 mRNA in tissues of lung adenocarcinoma and A549/H460 cell lines. Another study also demonstrated that miR-203 inhibits DKK1 expression via binding to its mRNA in rat mesenchymal stem cells [96]. As miR-203 is found to be expressed in HFs, it can be presumed that miR-203 may inhibit DKK1, and thus activate Wnt/β-catenin signaling and promote hair growth via inducing anagen phase. Indeed, a study focusing on RNA-binding motif protein 28 (RBM28) showed that RBM28 promoted hair growth via modulating the activity of miR-203 in human HF organ cultures [188]. Furthermore, Ma et al. have revealed that miR-203 may regulate goat HF development [189]. miR-203a-3p is evidenced to inhibit SMAD1 in HFSC of Sprague Dawley (SD) rats [190], which positively influences on hair cycle as SMADs are known to inhibit HF differentiation and hair growth [2,210].

### 6.6. miR-218

miR-218 via its role in Wnt/β-catenin signaling represents a hot spot molecule in cancer studies, as dysregulation of this miRNA is strongly associated with different cancers [211,212,213,214,215,216]. Except for the cancers, miR-218-5p plays an essential role in the HF and skin development again through Wnt/β-catenin signaling by targeting and inhibiting SFRP2—the antagonist of this signaling pathway [76]. Indeed, miR-218-5p is evidenced to promote HF development by inhibiting SFRP2. Treatment with the miR-218-5p mimic resulted in hair-regrowth in C57BL/6 mice, although the outcome was not as good as in case of treatment with exosomes containing this miRNA. This can be explained by the presence of multiple miRNAs in exosomes [77]. Besides, much depends on the delivery systems of miRNA. Delivery vectors are more developed for in vitro transfection than in vivo transfection. Although miR-218-5p seems a very promising candidate for hair restoration, it is still unclear whether this particular miRNA is capable to solely induce hair restoration in AGA because the abovementioned studies were performed on shaved/depilated dorsal skin of mice or HF organ culture obtained from C57BL/6 mice [77] or rabbits [76]. Additionally, there was no measurement of DKK1 conducted after the treatment with miRNA in any of those studies regarding the influence of miR-218 on hair growth. Intriguingly, upregulated miR-218-5p decreased DKK1 secretion in rheumatoid arthritis-fibroblast-like synovial cells [153]. This result also supports the idea that miR-218 may be one of the best candidates for AGA therapy. Therefore, it would be prudent to study the impact of miR-218 on DKK1 levels in the mouse model of human AGA induced by DHT treatment [27].

## 7. Main Challenge: Delivery Systems

RNA therapeutics need an effective technique to be delivered to the target cells, avoiding in vivo nuclease-mediated degradation. This is one of the major challenges in miRNA-based treatment along with specificity, stability, immune activation, and toxicity in vivo and in vitro [217]. Local delivery can be simply performed with the injection of naked RNA, while for systemic delivery, an effective delivery system is required [218]. Nucleic acids as well as other macromolecules are typically encapsulated with nanoparticles that are often modified with polyethylene glycol, cholesterol, or other moieties, or a special ligand is added to advance the uptake by the cell membrane. The encapsulated RNA molecule is then endocytosed by the cell. The nanoparticle is degraded, and the nucleic acid molecule is released into the cytoplasm [219]. Besides the nanoparticles, polyethyleneimine (PEI)-based delivery of miRNA is evidenced to be an effective approach. It has been used successfully for delivering miR-145 and miR-33a molecules in mice models [220]. PEI is a positively charged organic polymer that efficiently forms a complex with anionic RNA and provides an effective transfection in cells. Although branched and linear PEIs are used as miRNA delivery systems, they have limitations as well, such as low transfection efficiency and cytotoxicity. Poly (lactide-co-glycolide) (PLGA) is an FDA-approved biodegradable drug delivery system, however, due to the hydrophobic property, miRNA delivery is less efficient. Among polymer delivery carriers, poly (amidoamine) dendrimers are characterized with high transfection efficiency [221]. For studying the effect of miR-218-5p on hair regrowth in mice, as a delivery system, in vivo jetPEI was used effectively [77]. Compared with the PEI, in vivo jetPEI is more effective and safer, although it is relatively costly. As a non-viral vector, typically, lipid-based modified nanocarriers are used, e.g., lipofectamine [188], invivofectamine, oligofectamine, etc. [222]. The study has revealed the beneficial properties of argininocalix [4] arene, that seems to be an effective delivery system for miRNA therapeutics [223,224]. A promising candidate is lipid-based nanoparticles (LNP), that consist of the same component as the cell membrane and promote the uptake process [225,226]. Remarkably, LNP-based delivery of miR-634 has successfully reduced tumor xenograft growth in mice [227]. Evidently, solid lipid nanoparticles (SLNs) successfully work for delivering miR-34a for cancer stem cell therapy [228]. Moreover, the COVID-19 mRNA vaccine is also packed with LNP [229]. Thus, LNP application for miRNA delivery seems to be realized in the nearest future. However, LNPs also have disadvantages, such as the requirement of ultra-low-temperature storage [229]. Except for the non-viral delivery methods, there are viral delivery miRNA carrier systems, e.g., adenoviral, retroviral [230], lentiviral, and bacteriophage-based virus-like particle vectors [231]. However, viral vectors have disadvantages as well, e.g., the phage vector has a low loading capacity and needs sufficient studies, while in the case of lentiviral vectors, random genomic integration might result in the insertional mutation [231]. In terms of non-viral delivery systems, the exosome-based method is one of the most promising [231], although the difficulty of preparation hinders its development [232]. In hair growth studies, mostly lipofectamine (in vitro) [7,76,177,189,190] and in vivo jetPEI [77] are used as delivery systems. Taken together, according to the present data, there are a number of effective delivery carriers for miRNAs, but along with the advantages, drawbacks such as stability, toxicity, localized delivery, and integrity of nucleic acid still exist.

## 8. Concluding Remarks and Future Directions

Collectively, DKK1 has a critical role in the development of human AGA. Inhibition of DKK1 levels in the balding area of the scalp in populations that are at high risk of developing AGA might prevent the further progression of this disorder. On the other hand, it might even overcome the problem of hair regrowth via stimulating Wnt/β-catenin signaling. Therefore, assessment of DKK1-targeting miRNAs that are expressed in human HFs and involved in the Wnt/β-catenin signaling pathway may lay the groundwork for developing strategies of promoting hair regeneration and treatment of AGA. Nevertheless, further studies are needed to validate the described phenomenon. Additionally, miR-103/107, miR-203, and miR-218 among other miRNAs might represent the attractive therapeutic candidates for further studies focusing on modulation of the Wnt/β-catenin signaling pathway via regulating DKK1. Prominently, the functional spectrum of miRNAs is wide. Although certain miRNAs are capable to inhibit the Wnt antagonist, they might also target other key molecules that are instrumental in the regulation of this pathway. miR-29a ideally exemplifies the described complexity [167,168]. Thus, in-depth studies are needed to select the optimal miRNA as a potential drug candidate. Furthermore, it is crucial to design such an approach extremely delicately as the Wnt/β-catenin pathway is implicated in a myriad of biological processes and the intervention might trigger unfavorable consequences. Thus, an approach that implies the application of selected miRNAs that are delivered in HFs makes sense. Ultimately, miRNAs implicated in Wnt/β-catenin signaling that target DKK1 should be the center of foci in further studies to elucidate their roles and to aid in advancing strategies of AGA treatment.

## Figures and Tables

**Table 1:** Recent studies referring to miRNAs that directly target DKK1 and the association with certain disorders/biological processes.

miRNA	Disorder/Biological Process	miR Level	DKK1 Relative Expression	miR Predicted Binding Site in 3′UTR of DKK1	Study Type	Ref.
miR-1-3p	Oral squamous cell carcinoma	↓	↑	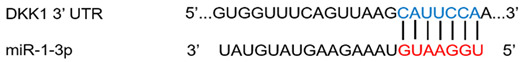	in vitro	[82]
miR-9-5p	Dopaminergic neuron differentiation	N/A	↓	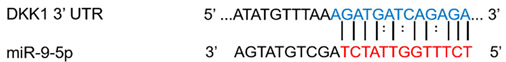	in vitro	[83]
miR-29a	Bone metabolism disorder	↓	↑	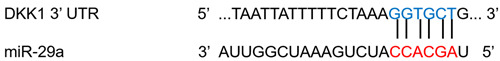	in vitro	[84]
miR-31	Breast cancer	↓	↑	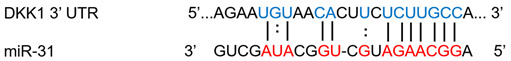	in vitro	[85]
miR-33a-5p	Esophageal cancer	↓	↑	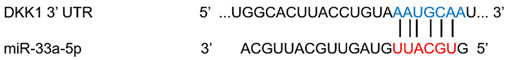	in vivo in vitro	[86]
miR-34a	Cardiac hypertrophy	↑	↓	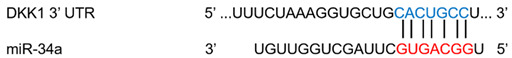	in vivo	[87]
miR-101-3p	Osteogenic differentiation	↑	↓	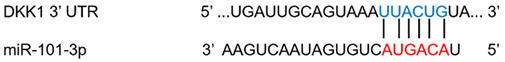	in vitro	[88]
miR-103a-3p	Osteogenic differentiation	↑	↓	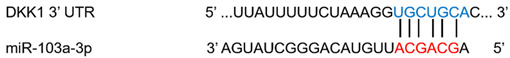	in vitro	[89]
miR-107	Osteosarcoma	↓	↑	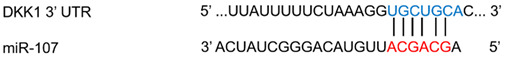	in vitro	[90]
miR-130b-3p	Melanoma	↑	↓	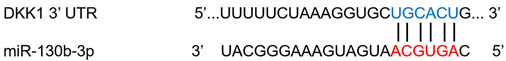	in vitro	[91]
miR-146a	Ankylosing spondylitis	↑	↓	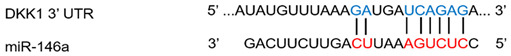	ex vivo	[92]
miR-152	Multiple myeloma	↓	↑	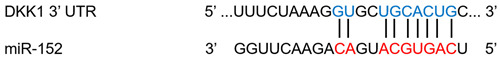	in vitro in vivo	[93]
*miR-186-5p	Idiopathic pulmonary fibrosis	↓	↑	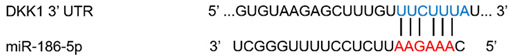	in vitro, ex vivo, in vivo	[94]
miR-203	Lung adenocarcinoma	↓	↑	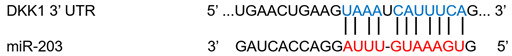	ex vivo, in vitro	[95]
	Osteoporosis		↑	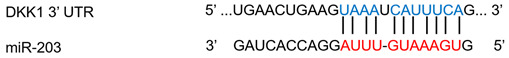	in vitro	[96]
miR-217	Colon cancer	↑	↓	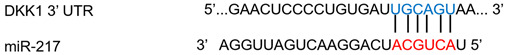	in vitro	[97]
	Hepatocellular carcinoma	↑	↓	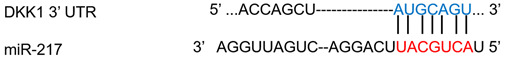	in vitro/ex vivo in vivo	[98]
				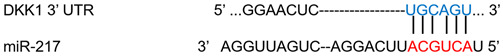		
	Osteonecrosis	↓	↑	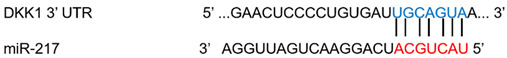	in vitro, ex vivo	[99]
miR-291a-3p	Osteoporosis	↓	↑	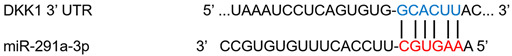	in vitro, in vivo	[100]
miR-302b-3p	Multiple myeloma	↓	↑	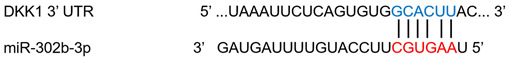	in vitro, in vivo	[101]
miR-302e	Cardiac hypertrophy	↑	N/A	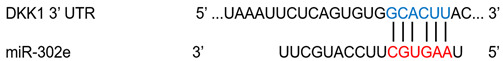	in vitro	[102]
	Cervical cancer	↓	↑	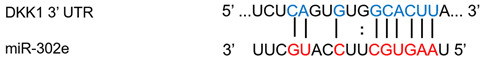	in vitro, in vivo	[103]
miR-335-5p	Diabetic osteoporosis	↓	↑	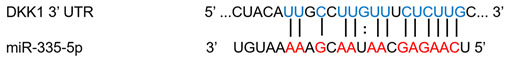	in vitro	[104]
miR-371/372/373	Stem cell tumorigenesis	↑	↓	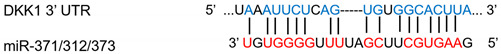	in vitro	[105]
miR-373-3p	Tongue squamous cell carcinoma	↑	↓	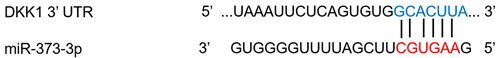	in vitro	[106]
miR-410	Colorectal cancer	↑	↓	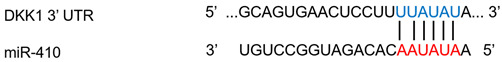	in vitro	[107]
miR-433-3p	Osteogenic differentiation	↑	↓	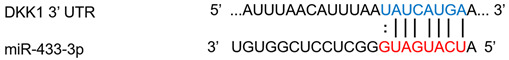	in vitro	[108]
miR-488	Fracture	↓	↑	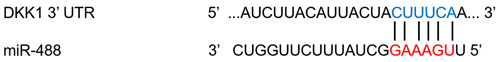	in vitro	[109]
miR-493-3p	Gastric cancer	↑	↓	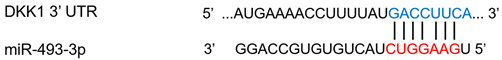	in vitro	[110]
miR-522	Hepatocellular carcinoma	↑	↓	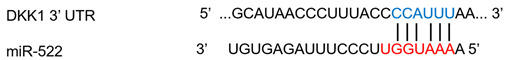	in vitro	[111]
miR-523-3p	Retinoblastoma	↑	↓	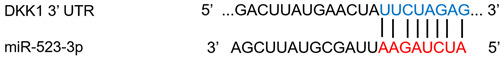	in vitro, ex vivo	[112]
miR-613	Rheumatoid arthritis	↓	↑	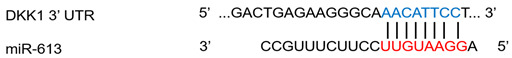	in vitro	[113]
miR-3064-3p	Cementoblast differentiation	↓	↑	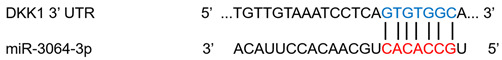	in vitro	[114]
miR-6783-3p	Lung adenocarcinoma	↑	↓	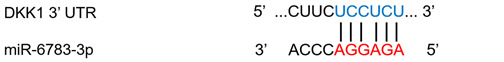	in vitro, in vivo	[115]
miR-6807-3p	Lung adenocarcinoma	↑	↓	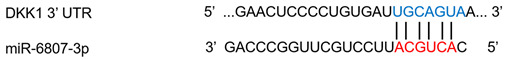	in vitro, in vivo	[116]
miR-BART10-3p	Gastric carcinoma	↑	↓	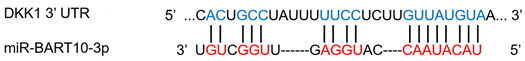	in vitro	[117]
miR-BART22	Gastric carcinoma	↑	↓	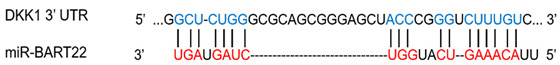	in vitro	[118]

Notes: Dots between miRNA and DKK1 sequence indicate wobble pairing. N/A, not applicable. *, These binding sites are cited from TargetScan.org.

**Table 2:** Studies focusing on miRNAs that are involved in hair growth and/or related signaling pathways and targeting proteins that are possibly implicated in hair growth regulation.

#	Study Title	miRNA	Influence on Hair Growth	Study Model	Type of Administration	Biological Function	Target Protein/Gene	Finding	Ref.
1	Differential expression of miR-let7a in hair follicle cycle of Liaoning cashmere goats and identification of its targets	miR-let7a	Regulatory	Goat skin samples, cell culture	in vitro transfection	Regulating FGF, IGF, C-Myc signaling pathways	IGF-1R, C-Myc, FGF5	miR-let7a regulates HF development via targeting C-myc, IGF-1R, and FGF5	[157]
2	Let-7b regulates alpaca hair growth by downregulating ectodysplasin A	miR-let-7b	Regulatory	Alpaca, cell culture	in vitro transfection	Inhibiting EDA signaling	EDA	miR-let-7b regulates hair growth via targeting EDA	[158]
3	Identification of microRNA-21 target genes associated with hair follicle development in sheep	miR-21	Regulatory	Sheep	in vitro transfection	Regulating CNKSR2-MAPK, KLF3, TNPO1-CCR2 signaling pathways	CNKSR2, KLF3, TNPO1	miR-21 targets CNKSR2, KLF3, and TNPO1 that might play a regulatory role in HF development	[159]
4	EZH2-mediated inhibition of microRNA-22 promotes differentiation of hair follicle stem cells by elevating STK40 expression	miR-22	Negative	Mouse, cell culture	in vitro transfection	Inhibiting MEF2-ALP signaling	STK40	miR-22 targets STK40 and inhibits the MEF2-ALP signaling pathway, and thus impedes the proliferation and differentiation of HFSC	[160]
5	Post-transcriptional regulation of keratinocyte progenitor cell expansion, differentiation and hair follicle regression by miR-22		Negative	Mouse, cell culture	in vitro transfection	Activating apoptotic signaling pathways	DLX3, FOXN1, HOXC13	miR-22 is a critical post-transcriptional regulator of the hair cycle and its activation results in hair loss	[161]
6	miR-24 affects hair follicle morphogenesis targeting Tcf-3	miR-24	Negative	Mouse, cell culture	in vitro transfection	Regulating Wnt/β-catenin signaling	TCF-3	miR-24 is expressed in the HF and it directly targets TCF-3, a regulator of the hair keratinocyte stemness	[162]
7	*miR*-24 controls the regenerative competence of hair follicle progenitors by targeting *Plk3*		Negative	Mouse, cell culture	in vitro transfection	Regulating PLK3-CCNE1 signaling	PLK3	miR-24 by targeting PLK3 limits the intrinsic growth competence of HF progenitor	[163]
8	miR-29 modulates Wnt signaling in human osteoblasts through a positive feedback loop	miR-29a	N/A	Cell culture	in vitro transfection	Activating Wnt/β-catenin signaling	DKK1, SFRP2, KRM2	miR-29a potentiates Wnt signaling via downregulation of the key antagonists of Wnt signaling, DKK1, KRM2, and SFRP2, which contributes to a gene expression program important for osteoblast differentiation	[164]
9	miR-29 suppression of osteonectin in osteoblasts: regulation during differentiation and by canonical Wnt signaling		N/A	Cell culture	in vitro transfection	Activating Wnt/β-catenin signaling	DKK1	miR-29a and Wnt signaling antagonist DKK1 expressions are negatively correlated	[165]
10	Protective effects of miR-29a on diabetic glomerular dysfunction by modulation of DKK1/Wnt/β-catenin signaling		N/A	Mouse, cell culture	Tail vain injection, in vitro transfection	Activating Wnt/β-catenin signaling	DKK1	miR-29a in diabetic mice attenuates the expression of DKK1 which inactivates Wnt/β-catenin signaling	[166]
11	miR-29a modulates tumor necrosis factor-α-induced osteogenic inhibition by targeting Wnt antagonists		N/A	Cell culture	in vitro transfection	Activating Wnt/β-catenin signaling	DKK1, GSK-3β	miR-29a plays an important role in regulating TNF-α-mediated osteogenic inhibition partly by targeting DKK1 and GSK-3β	[84]
12	LncRNA H19 overexpression activates Wnt signaling to maintain the hair follicle regeneration potential of dermal papilla cells		Positive	Mouse, cell culture	Subcutaneous injection, in vitro transfection	Activating Wnt/β-catenin signaling	DKK1, SFRP2, KRM2	lncRNA H19 induces miR-29a which activates Wnt signaling and induces HF regeneration in vitro and in vivo	[167]
13	miR-29a/b1 inhibits hair follicle stem cell lineage progression by spatiotemporally suppressing Wnt and BMP signaling	miR-29a/b1	Negative	Mouse, Cell culture	in vitro transfection	Inhibiting Wnt/β-catenin, BMP signaling pathways	LRP6, CTNNB1, BMPR1a, CCNA2	miR-29a/b1 overexpression causes hair loss by targeting Wnt and BMP	[168]
14	Chi-miR-30b-5p inhibits dermal papilla cells proliferation by targeting *CaMKIIδ* gene in cashmere goat	miR-30b-5p	Negative	Goat, cell culture	in vitro transfection	N/A	CaMKIIδ	Chi-miR-30b-5p targets CaMKIIδ and inhibits the proliferation of DP cells	[74]
15	miR-31-5p promotes proliferation and inhibits apoptosis of goat hair follicle stem cells by targeting RASA1/MAP3K1 pathway	miR-31	Positive	Cell culture	in vitro transfection	Activating MAPK signaling	RASA1	miR-31-5p suppresses apoptosis/promotes goat HFSC proliferation via inhibiting RASA1 and upregulating MAP3K1	[169]
16	Micro-RNA-31 controls hair cycle-associated changes in gene expression programs of the skin and hair follicle		Optimal balance	Mouse, cell culture	in vitro transfection	Balancing FGF, BMP, Wnt/β-catenin signaling	FGF10, SOST, BAMBI	miR-31 is involved in maintaining an optimal balance of gene expression in HFs via targeting a number of key molecules; thus, it has a positive influence on HF proper growth and development	[170]
17	MiR-31 promotes mammary stem cell expansion and breast tumorigenesis by suppressing Wnt signaling antagonists		N/A	Mouse, cell line	in vitro transfection	Activating Wnt/β-catenin, Inhibiting TGF-β, PRLR/STAT5 signaling pathways	DKK1, AXIN1, GSK-3β	miR-31 promotes Wnt/β-catenin signaling by targeting DKK1, thus, miR-31 represents a key regulator of breast tumorigenesis	[85]
18	Down-regulation of microRNA-31-5p inhibits proliferation and invasion of osteosarcoma cells through Wnt/β-catenin signaling pathway by enhancing AXIN1		N/A	Tumor samples cell culture	in vitro transfection	Activating Wnt/β-catenin signaling	AXIN1	miR-31-5p targets AXIN1, and via inhibiting the transcription of AXIN1, it activates Wnt/β-catenin signaling in OS cells	[171]
19	The major miR-31 target genes STK40 and LATS2 and their implications in the regulation of keratinocyte growth and hair differentiation		Negative	Mouse, cell culture	in vitro transfection	Promoting aberrant keratinocyte growth	STK40, LATS2	miR-31 targets a large number of gene expressions, including STK40 and LATS2, that play a role in keratinocyte growth and HF biology	[172]
20	Downregulation of LINC00707 promotes osteogenic differentiation of human bone marrow-derived mesenchymal stem cells by regulating DKK1 via targeting miR-103a-3p	miR-103a-3p	N/A	Cell culture	in vitro transfection	Activating Wnt/β-catenin signaling	DKK1	LINC00707 regulates the expression of DKK1 by targeting miR-103a-3p	[89]
21	MicroRNAs-103/107 regulate autophagy in the epidermis	miR-103/107	Positive	Mouse, cell culture	Subcutaneous injection	Activating PKC signaling	PLD1, PLD2	miR-103/107 may play an important role in maintaining the stemness of HFSCs and its levels may decrease with age	[173]
22	In vitro effect of microRNA-107 targeting Dkk-1 by regulation of Wnt/β-catenin signaling pathway in osteosarcoma	miR-107	N/A	OS tissue, cell culture	in vitro transfection	Activating Wnt/β-catenin signaling	DKK1	miR-107 inhibits the occurrence and development of OS by downregulating DKK1 through the Wnt/β-catenin signaling pathway	[90]
23	miR-124 promotes neural differentiation in mouse bulge stem cells by repressing Ptbp1 and Sox9	miR-124	Positive	Mouse, cell culture	in vitro transfection	Promoting HFSCs neuronal differentiation	SOX9, PTBP1	miR-124 promotes HFSCs neuronal differentiation via targeting SOX9 and PTBP1	[174]
24	miR-125/CDK2 axis in cochlear progenitor cell proliferation	miR-125	Negative	Rat, cell culture	in vitro transfection	Inhibiting CDK pathway	CDK2	miR-125 inhibits the proliferation of CPC by downregulating CDK2	[175]
25	Chi-miR-130b-3p regulates Inner Mongolia cashmere goat skin hair follicles in fetuses by targeting Wnt family member 10A	miR-130b-3p	Negative	Cell culture	in vitro transfection	Inhibiting Wnt/β-catenin signaling	Wnt10a	The study suggests that Wnt10a is a target gene of chi-miR-130b-3p. Thus, chi-miR-130b-3p may regulate epithelial cell and dermal fibroblast proliferation	[176]
26	Expression profiling and functional characterization of miR-26a and miR-130a in regulating Zhongwei goat hair development via the TGF-β /SMAD pathway	miR-130a miR-26a	Negative (miR-130a) Positive (miR-26)	Goat, cell culture	in vitro transfection	Regulating TGF-β/SMAD pathway	SMAD5, SMAD1	miR-26a and miR-130a regulate the HF development and growth through implication in TGF- β/SMAD signaling pathway	[177]
27	miRNA microarray profiling in patients with androgenic alopecia and the effects of miR-133b on hair growth	miR-133b	Negative	AGA scalp, cell culture	in vitro transfection	Inhibiting Wnt/β-catenin signaling	ALP, β-catenin, versican	miR-133b is abnormally highly expressed in patients with AGA. miR-133b may inactivate the Wnt/β-catenin pathway, and thus regulate hair growth	[150]
28	miR-140-5p in small extracellular vesicles from human papilla cells stimulates hair growth by promoting proliferation of outer root sheath and hair matrix cells	miR-140-5p	Positive	Cell culture	in vitro transfection	Inhibiting BMP signaling	BMP2	miR-140-5p plays a critical role in hair growth and cell proliferation and it represents a therapeutic target for alopecia	[178]
29	Preliminary study on microR-148a and microR-10a in dermal papilla cells of Hu sheep	miR-148amiR-10a	N/A	Cell culture	in vitro transfection	Inhibiting BMP signaling, regulating TGF-β/Smads signaling	BMP7	miR-148a and miR-10a inhibits the proliferation of Hu sheep dermal papilla cells	[179]
30	MicroRNA-148b promotes proliferation of hair follicle cells by targeting NFAT5	miR-148b	Positive	Cell culture	in vitro transfection	Activating Wnt/β-catenin signaling	NFAT5, Wnt10b	miR-148b activates the Wnt/β-catenin signaling pathway by targeting *NFAT5* and promotes the proliferation of human HF cells	[180]
31	miR-149-5p regulates goat hair follicle stem cell proliferation and apoptosis by targeting the CMTM3/AR axis during superior-quality brush hair formation	miR-149-5p	Positive	Goat, cell culture	in vitro transfection	Regulating AR transcriptional activity	CMTM3	miR-149-5p suppresses HF stem cell apoptosis by inhibiting CMTM3 and regulates the high-quality hair formation in goats	[181]
32	Downregulation of MicroRNA-152 contributes to high expression of DKK1 in multiple myeloma	miR-152	N/A	Mouse, cell culture	Femur injection, in vitro transfection	Activating Wnt/β-catenin signaling	DKK1	miR-152 blocked DKK1 transcriptional activity by binding to the 3′UTR of DKK1 mRNA. miR-152 is a master regulator in the pathobiology of MM via targeting DKK1	[93]
33	The effect of the microRNA-183 family on hair cell-specific markers of human bone marrow-derived mesenchymal stem cells	miR-182	Positive	Cell culture	in vitro transfection	N/A	N/A	miR-182 plays a key role in hair cell differentiation	[182]
34	microRNA-183 is involved in the differentiation and regeneration of Notch signaling-prohibited hair cells from mouse cochlea	miR-183	Positive	Mouse, cell culture, organ culture	in vitro transfection	Regulating Notch signaling	N/A	Inhibition of the Notch signaling pathway induces miR-183 expression, which participates in hair cell differentiation and regeneration	[183]
35	microRNA-183 is essential for hair cell regeneration after neomycin injury in zebrafish	miR-183miR-182 miR-96	Positive	Zebrafish	Micro injection	N/A	N/A	miR-183 cluster has a crucial role in the regeneration of hair cells in zebrafish larvae and it can be a novel target for hair cell regeneration	[184]
36	The intragenic mRNA-microRNA regulatory network during telogen–anagen hair follicle transition in the cashmere goat	miR-195	Positive	Goat	N/A	Regulating Wnt/β-catenin signaling	SMAD2 FZD6	miR-195 plays a crucial role in the regulation of HF initiation	[185]
37	miR-195-5p regulates hair follicle inductivity of dermal papilla cells by suppressing Wnt/β-catenin activation	miR-195-5p	Negative	Scalp specimen, cell culture	in vitro transfection	Inhibiting Wnt/β-catenin signaling	LRP6	miR-195-5p inhibits Wnt/β-catenin activation by targeting LRP6	[186]
38	The microRNA-200 family coordinately regulates cell adhesion and proliferation in hair morphogenesis	miR-200	Positive	Mouse, cell culture	in vitro transfection	Regulating Hippo/Yap signaling	CCNG2, CFL2, SNAI2, SEC23A, YWHAB, FAT1, PTK2, CDC42, CRK, ROCK2, WASL, ACTN1	miR-200 family has a critical function in mammalian skin development via regulation of cell adhesion and orientation in the hair germ	[187]
39	miR-203 is involved in osteoporosis by regulating DKK1 and inhibiting osteogenic differentiation of MSCs	miR-203	N/A	Human, cell culture	in vitro transfection	Activating Wnt/β-catenin signaling	DKK1	miR-203 by regulating DKK1 expression promotes the differentiation of rat MSCs into osteoblast-like cells	[96]
40	Overexpression of miR-203 increases the sensitivity of NSCLC A549/H460 cell lines to cisplatin by targeting Dickkopf-1		N/A	Human advanced lung adenocarcinoma tissue, cell culture, mouse	Subcutaneous injection, in vitro transfection	Activating Wnt/β-catenin signaling	DKK1	miR-203 by targeting the 3’UTR of DKK1 increases cisplatin sensitivity in A549/H460 cell lines	[95]
41	RBM28, a protein deficient in ANE syndrome, regulates hair follicle growth via miR-203 and p63		Positive	Cell culture, organ culture	in vitro transfection	N/A	P63	RBM28 directly regulates hair growth by ameliorating the expression or activity of miR-203 that inhibits transcription factor p63	[188]
42	Expression of miRNA-203 and its target gene in hair follicle cycle development of Cashmere goat		Regulatory	Goat, cell culture	in vitro transfection	N/A	DDOST, NAE1	miR-203 significantly downregulates the expression of DDOST and NAE1 and regulates the HF development in Cashmere goats	[189]
43	miR-203a-3p promotes loureirin A-induced hair follicle stem cells differentiation by targeting Smad1		Positive	Rat, cell culture	in vitro transfection	Inhibiting BMP signaling	SMAD1	miR-203a-3p inhibits SMAD1 and promotes HFSCs differentiation	[190]
44	The functions of ocu-miR-205 in regulating hair follicle development in Rex rabbits	miR-205	Negative	Rabbit	Intradermal injection	Regulating PI3K/Akt, Wnt/β-catenin, Notch, BMP signaling pathways	NOG	ocu-miR-205 promotes the apoptosis of DP cells via inhibiting the expression of genes involved in the PI3K/Akt, Wnt, and Notch signaling pathways, and activates the BMP signaling pathway	[191]
45	MiR-214 regulates the human hair follicle stem cell proliferation and differentiation by targeting EZH2 and Wnt/β-catenin signaling way in vitro	miR-214	Negative	Scalp tissue, cell culture	in vitro transfection	Regulating Wnt/β-catenin signaling	EZH2	Overexpression of miR-214 decreases the expression of EZH2, β-catenin, and TCF-4, while downregulation of miR-214 promotes the proliferation and differentiation of HFSCs	[192]
46	MicroRNA-214 controls skin and hair follicle development by modulating the activity of the Wnt pathway		Negative	Mice, cell culture	in vitro transfection	Inhibiting Wnt/β-catenin signaling	β-catenin	miR-214 regulates the Wnt signaling pathway and β-catenin expression in the developing and postnatal skin and HFs	[75]
47	DNMT1-mediated methylation inhibits microRNA-214-3p and promotes hair follicle stem cell differentiate into adipogenic lineages		Negative	Scalp tissue, cell culture	in vitro transfection	N/A	DNMT1, MAPK1	Expression of DNMT1, MAPK1, and miR-214-3p in HFSc are negatively correlated. DNMT1 promotes adipogenesis of HFSc by mediating miR-214-3p/MAPK1/p-ERK1/2 axis	[193]
48	Dermal exosomes containing miR-218-5p promote hair regeneration by regulating β-catenin signaling	miR-218-5p	Positive	Mouse, cell culture	Subcutaneous injection	Activating Wnt/β-catenin signaling	SFRP2	miR-218-5p ameliorates HF development by downregulating SFRP2 and promoting β-catenin. miR-218-5p-overexpressed exosomes lead to the onset of anagen	[77]
49	miR-218-5p regulates skin and hair follicle development through Wnt/β-catenin signaling pathway by targeting SFRP2		Positive	Rabbit, cell culture	in vitro transfection	Activating Wnt/β-catenin signaling	SFRP2	miR-218-5p enhances the Wnt signaling pathway by targeting SFRP2 and induces HF development	[76]
50	Osteogenic differentiation of fibroblast-like synovial cells in rheumatoid arthritis is induced by microRNA-218 through a ROBO/Slit pathway		N/A	Synovial tissues	in vitro transfection	Activating Wnt/β-catenin signaling	ROBO1	miR-218 promotes the osteogenic differentiation of rheumatoid arthritis fibroblast-like synovial cells by targeting ROBO1 and suppressing DKK1	[153]
51	Defining microRNA signatures of hair follicular stem and progenitor cells in healthy and androgenic alopecia patients	miR-324-3p	Positive	Scalp sample, cell culture	in vitro transfection	Regulating MAPK, TGF-β signaling pathways	REL A, HSP A2, MAPK1/3, TGF-β3	miR-324-3p regulates pathways implicated in HF growth and development	[194]
52	MiR-92a-1-5p and miR-328-3p are upregulated in skin of female pattern hair loss patients	miR-328-3p miR-92a-1-5p	Negative	Human	N/A	Implicating in multiple signaling pathways that are involved in FPHL	N/A	miR-92a-1-5p and miR-328-3p are involved in many signaling pathways and have a negative effect on FPHL	[195]
53	LncRNA-PCAT1 maintains characteristics of dermal papilla cells and promotes hair follicle regeneration by regulating miR-329/Wnt10b axis	miR-329	Negative	Scalp sample, mouse, cell culture	in vitro transfection	Inhibiting Wnt/β-catenin signaling	Wnt10b, ALP, BMP2, Versican, NCAM	PCAT1 promotes DP cells’ proliferation via activating Wnt/β-catenin signaling, while miR-329 negatively affects DP cells	[196]
54	Chi-miR-370-3p regulates hair follicle morphogenesis of Inner Mongolian cashmere goats	miR-370-3p	Positive	Goat, cell culture	in vitro transfection	Inhibiting TGF, FGF signaling	TGF-βR2, FGFR2	Chi-miR-370-3p inhibits the proliferation of epithelial cells and dermal fibroblasts via targeting FGFR2 and TGF-βR2, as well as induces HF morphog1enesis	[197]
55	Differential expression analysis of balding and nonbalding dermal papilla microRNAs in male pattern baldness with a microRNA amplification profiling method	miR-410 miR-221 miR-125b miR-106a	Negative	Human, cell culture	N/A	N/A	N/A	miR-221, miR-125b, miR-106a, and miR-410 are significantly upregulated in balding papilla cells and they may participate in the pathogenesis of male pattern baldness	[151]
56	LncRNA-XIST promotes dermal papilla induced hair follicle regeneration by targeting miR-424 to activate hedgehog signaling	miR-424	Negative	Mouse, cell culture	in vitro transfection	Inhibiting SHH signaling	ALP, Versican, NCAM, GLI1/2	miR-424 is sponged by XIST, which promotes SHH signaling and facilitates HF regeneration	[198]

Abbreviations: HFSC, hair follicle stem cells; PKC, protein kinase C; OS, osteosarcoma; PLD, phospholipase D; MM, multiple myeloma; CMTM3, CKLF-like MARVEL transmembrane domain-containing 3; AR, androgen receptor; FPHL, female pattern hair loss; CPC, cochlear progenitor cells; PI3K, Phosphatidylinositol 3′-kinase; NOG, noggin; TGF-βR2, transforming growth factor-beta receptor 2; FGF, fibroblast growth factor; FGFR, fibroblast growth factor receptor; EDA, ectodysplasin A; IGF, insulin-like growth factor; IGF-IR, type 1 insulin-like growth factor receptor; CNKSR2, connector enhancer of kinase suppressor of Ras 2; MAPK, mitogen-activated protein kinase; MAP3K1, mitogen-activated protein 3 kinase 1; KLF3, kruppel-like factor 3; TNPO, transportin; CCR2, chemokine receptor type 2; MEF2, myocyte enhancer factor-2; ALP, alkaline phosphatase; STK40, Serine/Threonine Kinase 40; DLX3, distal-less homeobox 3; FOXN1, forkhead box N1; HOXC13, Homeobox C13; TCF, T cell factor; PLK3, polo-like kinase 3; DKK1, dickkopf-related protein 1; SFRP2, secreted frizzled-related protein 2; KRM2, kremen 2; GSK-3β, glycogen synthase kinase 3β; LRP, low-density lipoprotein receptor-related protein; CTNNB1, catenin beta 1; BMPR1a, bone morphogenetic protein receptor 1 a; CCNA, cyclin-A2; CaMKIIδ, Ca2+/calmodulin-dependent protein kinase II δ; RASA1, RAS P21 protein activator 1; SOST, sclerostin; BMP, bone morphogenetic protein; BAMBI, BMP and activin membrane-bound inhibitor; PRLR, prolactin receptor; STAT5, signal transducer and activator of transcription 5; LATS2, large tumor-suppressor kinase 2; SOX9, SRY (sex determining region Y) box 9 protein; PTBP1, polypyrimidine tract-binding protein 1; CDK, cyclin-dependent kinase; Wnt, wingless and integrated-1; NFAT5, nuclear factor of activated T cells type 5; FZD6, frizzled class receptor 6; CCNG2, cyclin G2; CFL2, cofilin 2; SNAI2, snail family transcriptional repressor 2; SEC23A, SEC23 homolog A; FAT1, FAT atypical cadherin 1; PTK2, protein tyrosine kinase 2; CDC42, cell division cycle 42 homolog; ROCK2, Rho-associated coiled-coil containing protein kinase 2; P63, tumor protein 63; ACTN1, alpha-actinin-1; MSCs, mesenchymal stem cells; RBM28, RNA-binding motif protein 28; ANE, alopecia–neurological defects–endocrinopathy; EZH2, enhancer of zeste homolog 2; DNMT1, DNA methyltransferase 1; FGFR2, fibroblast growth factor receptor 2; GLI, glioma-associated protein; lncRNA, long noncoding RNA; XIST, X-inactive-specific transcript; SHH, sonic hedgehog; N/A, not applicable.

## Data Availability

Not applicable.
